# The Enhanced Ability of Peripheral Mononuclear Cells Differentiating into Neural Cells in Term Infants with Good Improvement Suffering from Severe Hypoxic Ischemic Encephalopathy

**Published:** 2014-07-27

**Authors:** Wei dong, Zhang Yuwen, Gong Xiaohui

**Affiliations:** 1 Department of pediatrics, Tongji University; 2 Department of Neonatology, Shanghai Children’s Hospital, Shanghai Jiao Tong University, Shanghai, China

**Keywords:** Blood Cells, Hypoxia-Ischemia, Brain, Infant, Newborn, Neurons

## Abstract

***Objective:*** It has been found that asphyxia influences proliferation and differentiation of brain neural stem cells in newborn animal models, and that peripheral blood stem cells play an important role in repairing brain damage. But it has not been reported yet whether asphyxia influences peripheral blood stem cells differentiating into neural cells, and whether with the progress of the disease there is a change of peripheral blood stem cells differentiating into neural cells in newborns with hypoxic ischemic encephalopathy (HIE).

***Methods:*** Fifty term HIE infants were enrolled in research from March, 2007 to March, 2010. There were 10 cases of the severe HIE patients with good improvement, the severe HIE patients with poor improvement, the moderate HIE patients, the mild HIE patients and the controls, respectively. The peripheral mononuclear cells collected within 24 hours and on 7th day after birth were cultured in vitro for 10 days to differentiate into neural cells. The induced nestin positive cells were identified with Immunohistochemistry and counted**.**

***Findings*** ***:*** Within 24 hours after birth, there were no difference of induced nestin positive cells among the severe HIE patients with good improvement (68.99±7.85), the severe HIE patients with poor improvement (71.43±6.88), the moderate HIE patients (73.34±6.46), the mild HIE patients (70.46±6.66) and the controls (71.13±7.19, F=0.51, *P*=0.7). In the severe HIE patients with obvious improvement, the induced nestin positive cells from 7th day peripheral blood mononuclear cells (94.50±15.57) increased markedly compared with that within 24 hours (68.99±7.85,* t*=4.66, *P*<0.001), and were higher than the induced nestin positive cells from 7^th^ day peripheral blood mononuclear cells in the severe HIE patients with no obvious improvement (94.50±15.57 vs 69.48±5.32, *t*=4.62, *P*<0.001).

***Conclusion:*** The ability of peripheral mononuclear cells differentiating into neural cells in term infants with good improvement suffering from severe HIE was enhanced, which may suggest possible relationship between the brain repair and the peripheral stem cells.

## Introduction

Neonatal hypoxic ischemic encephalopathy (HIE) is one of the most common causes of death and long-term neurological impairment in full-term neonates worldwide. Up to now, there is no specific effective treatment for the severe HIE. The potential use of stem cells to reduce brain damage is a possibility^[^^1^^]^. It has been found that asphyxia influences proliferation and differentiation of brain neural stem cells in newborn animal models^[^^2^^-^^4^^]^, and that peripheral blood stem cells play an important role in repairing brain damage^[^^5^^]^. But It has not been reported yet whether asphyxia influences peripheral blood stem cells differentiating into neural cells, and whether with the progress of the disease there is a change of peripheral blood stem cells differentiating into neural cells in newborns with HIE. This research will focus on these questions.

## Subjects and Methods

Fifty term newborn infants from the pediatric department of Shanghai Tongji hospital affiliated to Tongji University, Shanghai, China, were enrolled into the study from March 2007 to March 2010. 72 hours after birth, the severity of HIE was evaluated according to The Sarnat Grading Scale of HIE, and 20 serious cases, 10 moderate cases, and 10 mild cases were identified. All the patients met the HIE diagnostic criteria. 10 severe cases improved obviously 14 days after birth, which were defined as no consciousness, no seizure, no abnormal muscle tone, no brain stem symptom, whereas 10 severe cases poorly improved, which were defined as having one or more of the above manifestations 14 days after birth or died. 2 cases died within 1 week, and 6 cases died after 1 week. 10 newborn infants with wet lung acted as controls, presenting with breath difficulty, but oxygen saturation was normal. The study protocol was reviewed and approved by the local institutional ethics committee and written informed consent was obtained from all guardians prior to the study

**Blood sample collection:**

5 ml peripheral blood was collected from all newborn infants within 24 hours after birth. Again 5 ml blood was collected from severe HIE patients on 7^th^ day after birth if the patients were still alive. The mononuclear cells were separated and collected with Ficoll separating medium (GIBCO ) 1500r/min×5min, re-suspended in proliferation culture medium (see below), which were applied in the following process. 

**The proliferation culture of peripheral mononuclear cells (PMCs)** ^[^^6^^]^ **:**

Proliferation culture medium was prepared, which consisted of DMEM culture medium (GIBCO), 10% fetal bovine serum (FBS, GIBCO), penicillin (Shanghai new pioneer pharmaceuticals company) 100U/ml, streptomycin (Northern China pharmaceuticals company) 100µg/ml. PMCs were plated into 6 well culture-plates at the density of 1×10^7^ cells/ml with volume of 2 ml per well, cultured for 3 days in a humidiﬁed atmosphere (37 °C, 5% CO_2_).

**The differentiation culture of peripheral mononuclear cells (PMC)** ^[^^6^^]^ **:**

After proliferation culture of peripheral mononuclear cells for 3 days, the culture medium was eliminated, and sterilized PBS was added, then PMCs were gently repeatedly beaten by pipette so that the unattached cells could be eliminated. 2 wells were again continually added with proliferation culture medium as negative controls, and the other 4 wells were added with changed culture medium, namely differentiation culture medium, which consisted of DMEM/F12 (GIBCO), 10% fetal bovine serum (GIBCO), penicillin (Shanghai new pioneer pharmaceuticals company) 100U/ml, streptomycin (Northern China Pharmaceuticals Co) 100µg/ml, all trans retinoid acid (Sigma Co) 0.5 µM, nerve growth factor (Sigma Co) 100ng/ml, insulin (Bo-Yun Biotech) 25 µg/ml, transferring (Bo-Yun Biotech) 100µg/ml, heparin (Wanbang Pharmaceuticals Co) 2 µg/ml, progesterone (Shanghai Pharmaceutical Co Ltd) 60 nM. PMCs were cultured in a humidiﬁed atmosphere (37°C, 5% CO_2_), and half volume of culture medium in each well was renewed with the same medium with the original medium in the well (Proliferation culture medium or differentiation culture medium) every 3 days. The cell shape and growth were daily observed under inverted phase contrast microscope. On the 10th day of differentiation culture, the immunohistochemistry was carried out to identify the differentiated cells.

**Immunohistochemistry identification:**

Cells in each well were fixed with 4% paraform for 25 min, washed thoroughly with PBS 5 times, permeabilized with 1g/L Triton for 15 min, again washed thoroughly with PBS 5 times, rinsed with 3% H_2_O_2_ for 10 min, washed with PBS 5 times. 

**Table 1:** The clinical data of patients with neonatal hypoxic ischemic encephalopathy (HIE)

**Group (No)**	**Controls ** **(n=10)**	**Mild HIE ** **(n=10)**	**Moderate ** **HIE (n=10)**	**Serious HIE ** **With good ** **Improveme** **nt (n=10)**	**Serious HIE ** **with poor ** **improvement ** **(n=10)**	**F (X2)**	***P.*** ** value**
**Time after birth**	11.8 (7.4)	8.3 (6.5)	13.8 (6.9)	13.2 (6.5)	9.8 (7.5)	1.12	0.4
**Gestation weeks**	38.6 (1.1)	39.1 (1.6)	39.1 (1.6)	39.1 (1.5)	39.1 (1.6)	0.20	0.9
**Birth weight**	333.3 (464.6)	3112.4 (362.7)	3233.2 (491.9)	3186.3 (536.0)	3241.1 (485.3)	0.29	0.9
**Sex Male** ** Female**	6 4	5 5	6 4	7 3	4 6	2.17	0.8
**Delivery Cesarean** ** vaginal**	7 3	6 4	5 5	4 6	3 7	3.94	0.5

Agent A (Normal horse serum) from immunohistochemistry kit was dropped into culture wells (Hangzhou Baitong Biotechnology Co Ltd), incubated and protected from light for 45 min to block antigens. 5μg/ml anti-Nestin antibody (R&D systems) was added, incubated and protected from light at 4℃ overnight. Each cell was rinsed and washed with PBS for 5 min×5 times, and agent B (biotin labeled second antibody) from immunohistochemistry kit was added, incubated and protected from light at room temperature for 60 min, rinsed and washed with PBS for 5min×5 times. Agent C (horseradish peroxydase complex) was added and incubated at room temperature for 15 min, washed with PBS 5 for 5 min×5 times. DAB was added into each well to react for 3 min, and then immediately washed with double distilled water thoroughly. Immunohistochemistry positive cells in each well further identified by cell shape, which had at least two long cell processes, were counted under microscope, and the average number of positive cells in 4 wells (added with differentiation culture medium) was taken to be the positive cells number.

**Statistical methods:**

SPSS13.0 for windows was adopted to analyze the data. The difference of Neuron marker nestin positive cells, time after birth, gestational weeks, birth weight among HIE newborns with different severity within 24 hr was analyzed by ANOVA, The difference of sex and delivery mode among HIE newborns with different severity was analyzed by chi-square test. The difference of Neuron marker nestin positive cells in the severe HIE newborns between different improvement groups on 7^th^ day was analyzed by* t *test.

## Findings

**Fig. 1:**
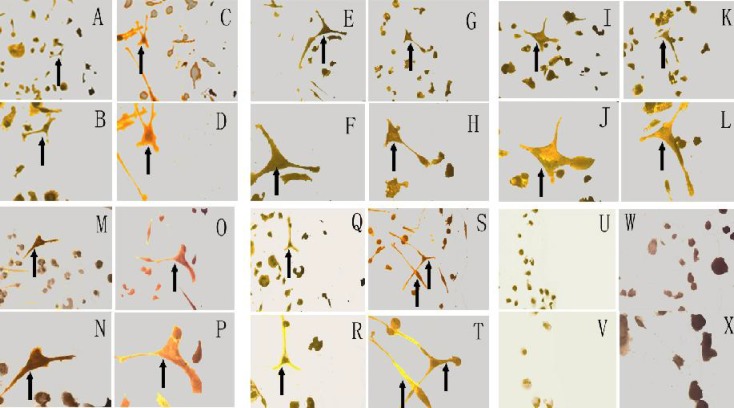
PMCs induced to differentiate into nestin positive cells

The patients’ clinical data was as follows (Table 1). There was no difference among groups as to sex, delivery, time after birth, gestational weeks and birth weight. All severe HIE patients received tracheal intubation and mechanical ventilation. After 10 days of cell culture in vitro, nestin positive cells with long cell processes could be identified among PMCs (Fig. 1). 

 The number of cells with neuron marker nestin had no difference among the mild HIE group, the moderate HIE group, the severe HIE with good improvement group, the severe HIE with poor improvement group and the controls (Table 2). The number of cells with neuron marker nestin differentiated from PMCs in the severe HIE with good improvement on 7th day was obviously higher than that within 24 hours after birth, and also higher than the number of cells with neuron marker nestin differentiated from PMCs in the severe HIE with poor improvement on 7^th^ day.

**Table 2:** Induced neuron marker nestin positive cells in HIE newborns within 24 hr after birth

**Group**	**Control (n=10)**	**Mild (n=10)**	**Moderate (n=10)**	**Severe with good improvement (n=10)**	**Severe with poor improvement (n=10)**	***F***	***P. value***
**Nestin Positive cells**	71.13 (7.19)	70.46 (6.66)	73.34 (6.46)	68.99 (7.85)	71.43 (6.88)	0.51	0.7

HIE: hypoxic ischemic encephalopathy

Note:“ * ” indicates nestin positive cells, DAB stain. Control: A (×200, <24h), B (×400, <24h), C (×200, 7d), D (×400, 7d); mild HIE: E (×200, <24h), F (×400, <24h), G (×200, 7d), H (×400, 7d) ; moderate HIE: I (×200, <24h), J (×400, <24h), K (×200, 7d), L (×400, 7d); Severe HIE with poor improvemnet: M (×200, <24h), N (×400, <24h), O (×200,7d), P (×400, 7d); Severe HIE with poor improvement: Q (×200, <24h), R (×400, <24h), S (×200, 7d), T (×400,7d); Negative control: U (×200, <24h), V (×400, <24h), W (×200, 7d), X (×400,7d)

The number of cells with neuron marker nestin in the severe HIE with poor improvement on 7th day was not different from that within 24 hours after birth (Table 3).

## Discussion

HIE is one of the main causes of death and long- term neurological impairment in full-term neonates. There is no specific effective treatment for the severe HIE. Many investigations have identified endogenous neural stem cells were mobilized when HIE took place, namely theneural stem cells in brain take part in HIE repair^[^^1^^]^, and there are also investigations revealing that peripheral blood stem cells have close relation to the damage repair of HIE brain^[^^5^^,^^7^^-^^11^^]^

 Umbilical mononuclear cells consist of multipotent stem cells^[^^12^^]^ and mesenchymal stem cells^[^^13^^]^ with neuron differentiation potential. Umbilical blood is a part of the neonatal peripheral blood, so it is likely that the neonatal peripheral blood cells possess similar character of the umbilical blood cells, namely consist of stem cells with neuron differentiation potential.

**Table 3:** Induced neuron marker nestin positive cells in the severe HIE newborns with different improvement on 7^th^ day after birth

**Group/Time**	**24 hr**	**7 d**	**t**	***P. *** **value**
**Severe HIE with good improvement (n=10)**	68.99 (7.85)	94.50 (15.57)	4.66	<0.001
**Severe HIE with poor improvement (n=8** * **)**	72.60 (7.27)	69.48 (5.32)	1.46	0.2
**t**	1.001	4.621		
***P. *** **value**	0.332	0.000		

* 8 cases: 2 cases died within 1 week, and the data on 7th day was absent, so the 2 cases were deleted when the data within 24 hrs after birth was analyzed only among the alive patients on 7^th^ day

Our data confirmed that PMCs from newborns and newborns with HIE could differentiate into cells with neural marker namely possessing neuron cells differentiation potential. Human peripheral CD14+ monocytes^[^^14^^]^ and CD133+ stem cells in umbilical blood^[^^15^^]^ can differentiate into cells with neural markers. It needs to be further confirmed which PMCs gradients in HIE newborn patients can differentiate into neuron cells. 

 The influence of asphyxia on neonatal peripheral blood stem cells differentiating into neuron cells has not yet been reported by now. Ischemia and anoxia have definite influence on neural stem cells in brain^[^^2^^-^^4^^,^^16^^,^^17^^]^. We could not find the influence of asphyxia on the ability of neonatal PMCs differentiating into neural cells, which may suggest the different influence of asphyxia on central neural stem cells and peripheral blood stem cells with neuron differentiation potential. 

 We found the ability of PMCs from severe HIE with good improvement on 7th day after birth to neural cells obviously increase, which may suggest these patients predominate at promoting neural cells differentiation, and may also suggest peripheral blood stem cells play a possible role in the HIE repair. The mechanism of peripheral blood stem cells taking part in central neural cells repair is unclear, which may relate to directly entering central nervous system to differentiate into neural cells to replace the injured brain tissues or to produce neurotrophin to promote the proliferation and differentiaton of brain internal neural stem cells^[^^18^^-^^27^^]^. The different differentiation ability of PMCs with the progress of HIE has close relationship with the prognosis. If the differentiation ability of PMCs can be identified as early as possible by improving the culture methods to shorten the culture time, it may be valuable to judge the prognosis. 

 The ability of peripheral mononuclear cells differentiating into neural cells in term infants with good improvement suffering from severe HIE was enhanced, which may suggest possible relationship between the brain repair and the peripheral stem cells. The mechanism of PMCs taking part in the protection of central nervous system injury suffered from asphyxia need to be further investigated.

## Conclusion

The ability of peripheral mononuclear cells differentiating into neural cells in term infants with good improvement suffering from severe hypoxic ischemic encephalopathy was enhanced, which may suggest possible relationship between the brain repair and the peripheral stem cells.
